# Ethical Leadership and Follower Moral Actions: Investigating an Emotional Linkage

**DOI:** 10.3389/fpsyg.2018.01881

**Published:** 2018-10-04

**Authors:** Yajun Zhang, Fangfang Zhou, Jianghua Mao

**Affiliations:** ^1^School of Management, Huazhong University of Science and Technology, Wuhan, China; ^2^School of Business Administration, Guizhou University of Finance and Economics, Guiyang, China; ^3^School of Business and Administration, Zhongnan University of Economics and Law, Wuhan, China

**Keywords:** ethical leadership, other-praising moral emotions, core self-evaluation, reporting unethical issues, unethical behavior

## Abstract

The effectiveness of ethical leadership has been extensively investigated. However, compared to the outcomes of ethical leadership, we still lack enough knowledge about the mechanisms underlying ethical leadership and its outcomes. Drawing from social information processing theory, this paper explores an emotional explanation for the effectiveness of ethical leadership. Adopting a time-lagged research design with responses from 64 leaders and 289 followers, the present research found that ethical leadership invokes followers’ other-praising emotions and eventually enhances their moral actions. Further, leader core self-evaluation contributes to the positive effects of ethical leadership on followers’ other-praising moral emotions and subsequent moral actions. Theoretical and practical implementations of these observations were discussed.

## Introduction

As ethical scandals are cropping up more frequently in recent times and in view of its unique effectiveness in modeling behavioral ethicality, ethical leadership is receiving greater research attention (Brown and Treviño, 2006; Demirtas and Akdogan, 2015). Characterized as a leadership demonstrating and promoting of “normatively appropriate conduct through personal actions and interpersonal relations” (Brown et al., 2005, p. 120), ethical leadership has been reported to have positive effects on a range of follower outcomes including task performance (Bouckenooghe et al., 2015), perceived leader effectiveness (Brown et al., 2005), organizational citizenship behavior (Piccolo et al., 2010), work place deviance (Resick et al., 2013), ethical behaviors (Mayer et al., 2009), and prosocial behaviors (Kalshoven et al., 2013). However, in spite of such empirical support, several researchers (Brown and Treviño, 2006; Bouckenooghe et al., 2015) have noted that our understanding of ethical leadership and its impacts on follower actions need to be improved due to the following reasons.

First, in comparison to the numerous outcomes of ethical leadership, little is known about the mechanisms through which ethical leaders trigger followers’ moral actions. Although the relationship between ethical leadership and follower ethical/unethical behaviors has been investigated (Mayer et al., 2009), we still lack enough research regarding why followers can translate their leaders’ ethical behaviors into their own moral actions, which is an equally essential part to understand ethical leadership effectiveness (van Knippenberg et al., 2004; Dinh et al., 2014). Thus,without investigating the mechanisms that drive the influence of ethical leadership on follower moral actions, we would not reach a comprehensive understanding about the effectiveness of ethical leadership.

Second, several scholars (Brown and Treviño, 2006; Chen and Hou, 2016; Zhang and Tu, 2016) have stressed the need to explore the boundary conditions of ethical leadership effectiveness. However, with few exceptions that had focused on follower characteristics such as self-esteem (Avey et al., 2011) or team climate (Chen and Hou, 2016), what mitigates or strengthens ethical leadership’s influence has remained undiscovered. Especially, little is known about whether and how the effectiveness of ethical leadership varies across different leader characteristics. Since paucity of information on the boundary conditions will limit the theoretical development and practical implications of ethical leadership (Brown and Treviño, 2006; Chen and Hou, 2016), it is necessary to examine such conditions to fully understand ethical leadership at the workplace.

The present research aims to address the gaps mentioned above. First, we rely on social information processing theory (SIP, Salancik and Pfeffer, 1978) to examine a moral emotional linkage between ethical leadership and follower moral actions. The emotional mechanism has been widely considered to be necessary to understand leader behaviors and to predict employee behaviors (Dasborough and Ashkanasy, 2002; Sadri et al., 2011). As in the moral domain, emotions consciously and unconsciously affect employees’ ethical behavior and ethical decision making (Greene and Haidt, 2002; Arsenio and Lemerise, 2004; Salvador and Folger, 2009; Harvey et al., 2016). However, research on ethical leadership has so far paid more attention to cognitive mechanisms such as (cognitive) trust (Xu et al., 2016), perceived accountability (Steinbauer et al., 2014) and perceived organizational politics (Kacmar et al., 2013), while research on leadership and business ethics has not done so with regard to the role that emotions play in employees’ reactions to ethical leadership (Brown and Mitchell, 2010). Although scholars have emphasized the vital role of moral emotions in translating moral standards into moral actions (see a review of Tangney et al., 2007; Lindebaum et al., 2017), few studies, as far as we know, have empirically examined this relationship.

Drawing from social information theory (Salancik and Pfeffer, 1978), which posits that environmental information cues shape individual’s attitudes and behaviors by indicating what a person’s attitudes and opinions should be, we propose that ethical leadership would invoke followers’ other-praising moral emotions, which eventually triggers followers to report more ethical issues and engage in less unethical behavior. Second, we believe that an ethical leader is more likely to evoke followers’ other-praising moral emotions when the leader has high core self-evaluation. Salancik and Pfeffer (1978) have pointed out that, as information cues become more salient, individual’s attitudes and behaviors are more likely to change. Hence, we believe that when an ethical leader has high core self-evaluation, he/she would be more confident about his/her own ethical beliefs and actions, making the ethical cues more salient to invoke followers’ other-praising moral emotions.

**FIGURE 1:**
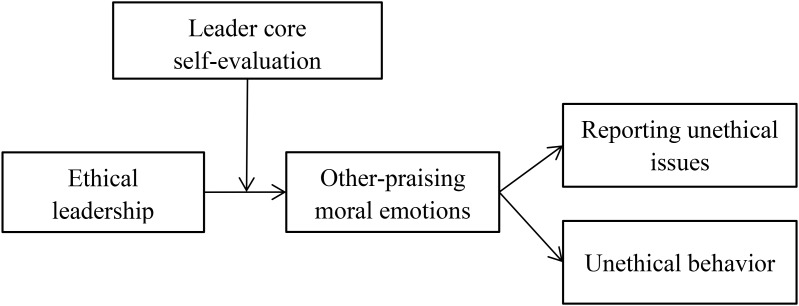
Theoretical model.

Our research contributes to ethical leadership and moral emotions literature in the following ways. First, by linking ethical leadership with follower moral actions through moral emotions, our research provides a fundamentally emotional explanation of why ethical leadership promotes follower moral actions. Second, by focusing on other-praising moral emotions, our research discusses how emotions in specific-domain (i.e., moral domain) can help explain the effectiveness of ethical leadership, thus contributing new insights to emotion literature. Finally, by examining the moderating role of leader core self-evaluation, the present research clarifies the potential boundary condition of ethical leadership effectiveness. Our theoretical framework is shown in **Figure 1**.

## Theory and Hypotheses

### SIP Theory

Individual’s job attitudes and behaviors are results of complex processes. Previous research has emphasized the vital role of need-satisfaction models in shaping employee’s job attitudes and behaviors. For example, Maslow (1943) proposed the hierarchy of needs model to explain how different needs guide individual’s specific behaviors. However, taking the social information processing perspective, Salancik and Pfeffer (1978) argue that the context and the consequences of past choices significantly influence individuals’ attitudes and behaviors, which go beyond the effects of individual predispositions and rational decision-making processes. Specifically, as social information processing theory posits, individual perceptions, attitudes and behaviors can be shaped by information cues, such as work requirements and expectations from the social environment (Salancik and Pfeffer, 1978; Bhave et al., 2010). Specifically, Gundlach et al. (2003) indicated that individuals’ translating of information cues could trigger individuals’ emotional reacts. For example, employees may experience anger emotion if they translate others’ whistle-blowing behavior to responsibility avoidance behavior. Meanwhile, several scholars have emphasized the vital role of emotion in processing information cues and in translating those cues into moral judgment and actions (e.g., Arsenio and Lemerise, 2004; Dodge and Rabiner, 2004).

According to SIP theory, one of the important sources of information is individuals’ immediate social environment, which has two general effects on individuals’ attitudes and behaviors (Salancik and Pfeffer, 1978). First, individuals’ social environment helps construct meaning directly through the guidance of socially acceptable beliefs, needs, attitudes, and reasons impinging on actions (Bhave et al., 2010). For example, leaders’ continuous statements about ethical standards and principles underlying work conditions force employees to either reject such statements or include them during employees’ own evaluations. Second, social influence and context focus individuals’ attention on certain specific information, which makes the information more salient, raises expectations, and highlights the logical consequences of individual behaviors (Salancik and Pfeffer, 1978). For example, coworkers may highlight the bad or unhealthy effects of their products to customers and state that their work was unethical when judged against the prevailing social norms.

According to social information processing theory, such environmental information cues help employees to construct and interpret events and shape their attitudes and behaviors by indicating what a person’s attitudes and opinions should be (Salancik and Pfeffer, 1978). Previous research has validated the work-related outcomes explained by SIP theory, such as procedural justices (Goldman, 2001), job satisfaction (O’Reilly and Caldwell, 1985), work-family conflict (Bhave et al., 2010), as well as leadership effectiveness (Chiu et al., 2016).

### Ethical Leadership and Other-Praising Moral Emotions

Ethical leadership is conceptualized as the “demonstration of normatively appropriate conduct through personal actions and interpersonal relationships, and the promotion of such conduct to followers through two-way communication, reinforcement, and decision-making” (Brown et al., 2005, p. 120). First, ethical leaders get legitimized by modeling normatively appropriate behaviors such as honesty, fairness and care. Second, ethical leaders not only pay attention to ethics themselves, but they also take specific actions to make ethics salient in the social environment, say, by communicating with followers about ethics, allowing followers to speak up their ideas or opinions (Bass and Steidlmeier, 1999), setting ethical standards and rewarding ethical conduct (Treviño et al., 2003). Finally, ethical leaders embed ethicality into their decision-making process by considering the ethical consequences of their decisions and making fair choices as a model for others (Bass and Avolio, 2000). Multiple studies have suggested that ethical leadership predicts followers’ work attitudes and behaviors, such as job satisfaction (Brown et al., 2005), psychological well-being (Avey et al., 2012), performance (Bonner et al., 2016), employee voice (Lee et al., 2017), OCB (Bonner et al., 2016; Wang and Sung, 2016) and misconduct (Mayer et al., 2010).

Moral emotions refer to the emotions that are linked to the “interests or welfare of society or at least of persons other than the judge or agent” (Haidt, 2003: 854), which typically include self-conscious emotions such as shame and guilt, other-condemning emotions such as anger and disgust, and other-praising emotions such as elevation and gratitude (Brown and Mitchell, 2010). Moral emotions have been thought to play a vital role in linking moral standards and moral behaviors (Tangney et al., 2007) because those emotions provide the motivational force (i.e., the power and the energy) for individual to do good and to avoid doing bad (Kroll and Egan, 2004). However, with very few exceptions that focus on the trait qualities of moral emotions (e.g., Eisenbeiss and Knippenberg, 2015), researchers have not empirically examined how the state qualities of moral emotions can help to explain the linkage between moral standards and moral behaviors.

In present research, we focus on other-praising moral emotions to answer how followers translate ethical leadership into their own moral behaviors. Other-praising moral emotions refer to the emotions that are positive and other-targeted, such as elevation, gratitude, and awe (Brown and Mitchell, 2010). We emphasize the mediating role of other-praising moral emotions for two reasons. First, ethical leaders stick to high ethical standards when making decisions (Lee et al., 2017), which will be more likely to invoke followers’ positive rather than negative moral emotions. Second, through communicating with followers about ethical issues and responding to followers’ suggestions, ethical leaders will be more possible to evoke followers’ leader-targeted rather than self-targeted moral emotions, such as elevation and gratitude.

Drawing on social information processing theory, we believe employees’ moral emotions and moral behaviors can be shaped by the ethical information cues (e.g., values, standards and behaviors) exhibited by their direct leader (Salancik and Pfeffer, 1978). Specifically, we propose that ethical leadership will evoke followers’ other-praising moral emotions due to the following reasons. First, we anticipate that ethical leadership can invoke follower moral emotions (e.g., elevation, inspiration) because ethical leaders express strong ethicality in their behaviors. Since the direct leader is one of the most important components of work environment for employees (Bass and Stogdill, 1990), the beliefs and behaviors of the direct leader provide the salient information cues that are capable of progressively changing employee attitudes and behaviors. Since ethical leaders exhibit high ethical beliefs and behaviors, followers will translate leaders’ ethical values and behaviors into their own feelings, e.g., generating the other-praising emotions such as elevation and awe.

Second, ethical exemplars encourage followers to praise moral emotions (e.g., elevation, awe) by demonstrating the desire for being just and helping others. Having been characterized thus as moral persons, ethical leaders start being seen not only as fair and principled decision-makers in organizations but also moral examples who care about the broader society (Brown and Treviño, 2006). By demonstrating self-sacrifice and self-transcendence (Mayer et al., 2012a), ethical leaders can easily invoke followers’ other-praising moral emotions. For example, when Martin Luther King was giving the famous speech “I Have a Dream”, the audience became charged with moral emotions such as elevation, inspiration, and awe. Third, ethical leaders elicit followers’ praising moral emotions (e.g., gratitude, inspiration) by taking care of followers’ needs and welfare. Haidt (2003) argued that, when an individual perceives that another person has done some good deeds for him/her, he/she will experience the emotion of gratitude. Thus, by continuously considering followers’ needs, ethical leaders will easily evoke praising moral emotions among their followers (Cropanzano et al., 2017).

Although the association between ethical leadership and followers’ other-praising moral emotions have not been directly examined, several previous findings could be seen to be providing supportive evidence for our proposed relationship. For example, Vianello et al. (2010) found that leaders’ self-sacrificing and interpersonal fairness elicited followers’ elevation. Similarly, Haidt (2003) pointed out that kindness and self-sacrifice are powerful elicitors of awe and elevation emotion. Therefore, we propose that:

Hypothesis 1: Ethical leadership is positively related to other-praising moral emotions.

### Other-Praising Moral Emotions and Moral Actions

The association between individual emotions and behavioral reactions has received much attention over the past few decades (Cropanzano et al., 2017; Lebel, 2017). For example, as cognitive appraisal theory posits (Lazarus, 1991), each discrete emotion predicts a specific action tendency (O’Leary-Kelly et al., 2017), e.g., anger predicts attack, compassion predicts helping, and anxiety predicts avoidance. In the moral emotion domain, although the linkage between each moral emotion and the specific behavioral tendency has not been fully revealed, research has made much progress in predicting behaviors via moral emotions. For example, Cropanzano et al. (2017) proposed that other-condemning moral emotions (anger, disgust, and contempt) invoked by leader-member exchange (LMX) differentiation will harm the LMX relationship in the future.

Since moral emotions are linked to the interests or welfare of society or of persons, moral actions will be more likely to become its behavior tendencies. As Kroll and Egan (2004) noted, moral emotions provide the motivational force—the power and energy—to do good and to avoid doing bad. In the present research, we propose that other-praising moral emotions positively affect followers’ moral actions to report unethical issues and to avoid doing unethical behaviors. First, other-praising moral emotions provide followers with more psychological power and energy to engage in moral actions (Kroll and Egan, 2004). Haidt (2003) pointed out that other-praising moral emotions (e.g., elevation and awe) “create a more generalized desire to become a better person oneself” (p. 861). Similarly, Algoe and Haidt (2009) argue that employees who are high in other-praising moral emotions should motivate changes and behaviors that are beneficial in the long run. Thus, when employees are charged with other-praising moral emotions, they will be more likely to engage in moral actions themselves, such as reducing unethical behaviors and reporting unethical issues for sustainable development.

Second, other-praising moral emotions broaden followers’ awareness and encourage followers to display more novel, exploratory and ethical behaviors. Fredrickson (1998) “broaden and build model” suggests that positive emotions prompt individuals to pursue novel, varied, and creative paths of actions rather than discard trivial behavioral scripts. Other-praising moral emotions fit well with this “broaden and build model”. Thus, employees with high other-praising moral emotions will go beyond their own normal duties to display more ethical behaviors. Furthermore, previous researchers have pointed to the positive relationship between other-praising moral emotions (e.g., elevation, gratitude, and admiration) and prosocial or ethical behaviors, such as helping others (Haidt, 2003; Algoe and Haidt, 2009). Therefore, we propose that:

Hypothesis 2: Other-praising moral emotions is positively related to followers’ moral actions, such as (a) reporting more ethical issues and (b) engaging in less unethical behavior.

Combining the above arguments—because ethical leadership acts as a critical antecedent of followers’ other-praising moral emotions (Hypothesis 1)—and because followers’ other-praising moral emotions could motivate them to do moral actions, we anticipate that other-praising moral emotions play a critical role in translating positive external influences (i.e., ethical leadership) to followers’ actual moral actions. Haidt (2003) also suggested that other-praising moral emotions encourage individuals to be a better person and to follow the moral example (i.e., the ethical leader) to demonstrate more ethical behaviors, creating “a virtuous ripple effect”. Hence, we argue that ethical leadership can invoke followers’ other-praising moral emotions, which in turn will lead to increased willingness to report unethical issues and decrease unethical behaviors at work.

Hypothesis 3: Ethical leadership will have positive effects on followers’ moral actions, such as (a) reporting more ethical issues and (b) engaging in less unethical behavior by invoking followers’ other-praising moral emotions.

### Moderating Effect of Leader’s Core Self-Evaluation

Although we believe that ethical leadership can invoke followers’ other-praising moral emotions, whether the followers would indeed be moved and inspired depends on the extent to which the followers treat their ethical leader as an important source of information. According to social information processing theory (Salancik and Pfeffer, 1978), an individual’s immediate social environment provides information cues to shape the individuals’ attitudes and behaviors. However, although the direct leader is one of the important environmental factors for employees, different leaders may influence their followers differently (Rees and Segal, 1984). For example, if an ethical leader has no confidence about what he/she believes and what he/she has done, the followers may question their leaders’ ethical behavior, let alone generating moral emotions.

This paper focuses on leader core self-evaluation as the boundary factor for several reasons. First, core self-evaluation provides an integrative framework addressing the effects of employee dispositions on their job attitudes (Bono and Judge, 2003). Judge et al. (2003) identified four components of core self-evaluation: generalized self-efficacy, self-esteem, emotional stability, and locus of control. Since these traits are fundamental and broaden self-perceptions, core self-evaluation is thought to have overarching influence on all other appraisals (Johnson et al., 2008). Second, core self-evaluation reflects people’s beliefs about their own ability to interact with the environment by exhibiting their own behaviors (Bono and Judge, 2003). By demonstrating efficacy beliefs while interacting with others, individuals with high core self-evaluation provide additional information cues for others to translate individuals’ behaviors. Third, previous leadership studies have called for investigating the role of core self-evaluation on leadership effectiveness (Resick et al., 2009). Hence, it is important to explore how the leader’s core self-evaluation affects the effectiveness of ethical leadership.

We propose that when the leader has high core self-evaluation, the positive effect of ethical leadership on followers’ other-praising moral emotions get strengthened. According to social information processing theory, employees’ attitudes and behaviors can be shaped by environment information cues (Salancik and Pfeffer, 1978). As a part of the followers’ immediate social environment, leader’s behaviors could exhibit both ethical and efficacy information. When an ethical leader has high core self-evaluation, he/she will be more confident about what he/she has done (Bono and Judge, 2003), thus making the ethical information (e.g., values, standards and behaviors) more salient for employees. On one hand, ethical leaders with high core self-evaluation will carry out more ethical standards and practices, thus invoking followers’ other-praising moral emotions by strengthening the ethical values and behaviors. On the other hand, high core self-evaluation leaders have high self-regulatory capacities to control their own actions to cope with external constraints (Johnson et al., 2008), which encourages followers to develop more positive feelings toward their leader. On the contrary, when an ethical leader has low core self-evaluation, he/she may have little confidence or ability to stick to ethical standards and behaviors himself/herself, thus conveying less ethical information cues to followers and invoking less moral emotions eventually. Therefore, we propose that:

Hypothesis 4: Leader core self-evaluation moderates the relationship between ethical leadership and other-praising moral emotions, such that the relationship between ethical leadership and other-praising moral emotions will be strengthened when leader has high core self-evaluation.

According to social information processing theory (Salancik and Pfeffer, 1978), leader’s ethical and efficacy characters could jointly affect employees’ attitude and behaviors. Since ethical leadership and leader core self-evaluation interact to influence employees’ other-praising moral emotions (Hypothesis 4) and other-praising moral emotions elicit moral actions (Hypothesis 2), we propose a mediated moderation effect to theorize that followers’ other-praising moral emotions help translate leader’s ethical and efficacy characteristics into their own moral actions.

Hypothesis 5: Other-praising moral emotions mediate the effect of interaction between ethical leadership and leader core self-evaluation on followers’ (a) reporting ethical issues and (b) unethical behavior.

## Methods

### Participants and Procedures

Data was collected from several organizations located in Mainland China. Industry of these companies varies from manufacturing, real-estate and high-tech industry. A time-lagged data collection method was designed to reduce the potential common method bias. At Time 1, 72 teams were contacted in these companies, ranging from research (29%), production (43%), sales (18%), and other functional teams (11%). Each team has one leader and more than three employees (the average number of followers per team is 4.52). One of the authors went directly to the workplace to distribute the questionnaire. Each participant was offered with a questionnaire, a $2 gift, and an introductory letter to briefly introduce the research purpose and ensure participants’ confidentiality. 40 min later, we collected the questionnaire back. Finally, we received questionnaires from 72 leaders and 350 followers. About 7 weeks later, at Time 2, we distributed the questionnaire directly to the person who participated in the first survey and a total of 64 leaders (a response rate of 89%) and 295 followers (a response rate of 84%) responded. After excluding some uncompleted questionnaire, we finally identified 64 leaders and 289 followers. 48% of leaders were women and their average age was 39.0 years (*SD* = 8.47), and they have worked in their company for an average of 9.9 years (*SD* = 9.4). 58% of followers were women and their average age was 31.73 years (*SD* = 8.41), and their average organizational tenure was 5.46 years (*SD* = 7.01).

At Time 1, followers were asked to rate ethical leadership and their demographics background information and leaders were asked to report their own core self-evaluation and demographics information. At Time 2, followers completed measures of moral emotions and reporting unethical issues and leaders rated their followers’ unethical behaviors.

### Measures

To ensure the internal validity of our translated scales, a back-translation process (c.f. Brislin, 1970) was conducted on all survey items.

#### Ethical Leadership

Ethical leadership was measured with a ten-item scale developed by Brown et al. (2005). Sample items include “(my leader) sets an example of how to do things the right way in terms of ethics” and “(my leader) “conducts his or her personal life in an ethical manner” (α = 0.96). Items were rated on a 7-point scale ranging from 1 (totally disagree) to 7 (totally agree).

#### Other-Praising Moral Emotions

We measured the five other-praising moral emotions proposed by Brown and Mitchell (2010), by using the format for assessment from Brunstein (1993). The five other-praising moral emotions are elevation, awe, inspiration, gratitude, and admiration. Followers were asked to report the extent they feel when interaction with their leader during the past months. Items were rated on a 7-point scale ranging from 1 (not at all) to 7 (very frequently). The Cronbach’s α was 0.86.

#### Leader Core Self-Evaluation

We measured leader core self-evaluation with 12-item from Judge et al. (2003). Sample items were “I am confident 1 get the success I deserve in life” and “I complete tasks successfully” (α = 0.94). Items were rated on a 7-point scale ranging from 1 (totally disagree) to 7 (totally agree).

#### Reporting Unethical Issues

Reporting unethical issues was measured with the two items from Mayer et al. (2013)’s reporting unethical conduct scale. The items are “If I personally observed conduct that violated our company’s standards of ethical business conduct I would report it” and “If I witnessed an employee violate our company’s code of conduct I would report it” (α = 0.90). Items were rated on a 7-point scale ranging from 1 (totally disagree) to 7 (totally agree).

#### Unethical Behavior

Leaders were asked to rate followers’ unethical behavior with a seven-item scale from Moore et al. (2012). Sample items include “falsifies a receipt to get reimbursed for more money than he/she spent on business expenses” and “takes property from work without permission” (α = 0.76). Items were rated on a 7-point scale ranging from 1 (never) to 7 (daily).

#### Control Variables

Since we based on social information processing perspective to examine how ethical leadership affects follower moral emotions, we controlled follower age, gender, and the interaction frequency between leader and follower (Lopes et al., 2005) to exclude potential confounded effects. Frequency of interaction with leader was measured with a 3-point item adapted from McAllister (1995) ranging from 1 (many times daily), to 3 (once or twice in the past 1 month). The item was “How frequently do you interact with your supervisor at work?” Follower gender was measured as a dummy variable, with “0” refers men and “1” refers to women.

### Analytical Strategy

Within-group interrater agreement (rwg, James et al., 1993) and ICC values were computed to examine whether employee-rating ethical leadership could be aggregated to team level. Ethical leadership had an average r_wg_ value of 0.94 with ICC(1) and ICC(2) of 0.46 and 0.80. Thus, according to these results, we aggregated ethical leadership to team level.

Follow the recommendation of Raudenbush et al. (2011), we used hierarchical linear modeling to test our hypotheses. As our hypothesis 2 and hypothesis 3 indicated cross-level indirect effect, we used Montel Carlo method to test those hypotheses (Preacher et al., 2010).

## Results

### Preliminary Analyses

**Table 1** provides the means, standard deviations, reliability, and correlations among all variables. Ethical leadership was positively related to moral emotions (*r* = 0.33, *p* < 0.001). Moral emotions was positively related to reporting unethical issues (*r* = 0.43, *p* < 0.001) and was negatively related to unethical behavior (*r* = −0.25, *p* < 0.001).

**Table 1:** Means, standard deviations, and correlations for all variables.

Variables	Mean	*SD*	1	2	3	4	5	6	7
1 Follower gender	0.58	0.50							
2 Follower age	31.50	7.96	−0.11						
3 Interaction frequency	1.38	0.61	−0.00	0.11					
4 Ethical leadership	6.07	0.93	−0.05	0.14^∗^	0.08				
5 Other-praising moral emotions	4.82	1.12	−0.03	0.02	0.01	0.33^∗∗∗^			
6 Reporting unethical issues	5.12	1.27	−0.02	0.06	0.04	0.34^∗∗∗^	0.43^∗∗∗^		
7 Unethical behavior	1.09	0.14	0.04	0.01	−0.05	−0.39^∗∗∗^	−0.25^∗∗∗^	−0.30^∗∗∗^	
8 Leader core self-evaluation	4.78	1.41	−0.07	0.01	−0.10	−0.04	−0.12^∗^	−0.14^∗^	0.05

*^∗^p < 0.05, ^∗∗∗^p < 0.001.*

We adopted the confirmatory factor analysis to verify discriminant validity of all the constructs. The measurement model was composed of four latent variables (ethical leadership, moral emotions, reporting unethical issues, and unethical behavior) with 24 indicators (10 items for ethical leadership, 5 items for moral emotions, 2 items for reporting unethical issues, and 7 items for unethical behavior). Results (see **Table 2**) showed that the four-factor model had the best fi to the data (χ^2^ = 1487.84, df = 485, χ^2^/ df = 3.07, CFI = 0.90, TLI = 0.89, RMSEA = 0.08), indicating that the constructs used in our model had good discriminant validity.

**Table 2:** Confirmatory factor analyses.

Models	χ^2^	Df	χ^2^/ df	CFI	TLI	RMSEA	Δχ^2^	*p*
Four-factor Model	622.60	246	2.53	0.92	0.91	0.07		
Three-factor Model^a^	1241.20	249	4.99	0.78	0.76	0.12	618.60	<0.001
Three-factor Model^b^	678.37	249	2.72	0.91	0.89	0.08	55.77	<0.001
Two-factor Model^c^	1296.03	251	5.16	0.77	0.75	0.12	673.43	<0.001
One-factor Model	1550.87	252	6.15	0.71	0.68	13	928.27	<0.001

*^a^In the three-factor model, items of ethical leadership and moral emotions were loaded on one factor. ^b^In the three-factor model, items of reporting unethical issues and unethical behavior were loaded on one factor. ^c^In the two-factor model, items of ethical leadership and moral emotions were loaded on one factor, items of reporting unethical issues and unethical behavior were loaded on one factor.*

### Hypotheses Testing

We used hierarchical linear modeling to test our hypotheses. As shown in **Table 3**, after controlling follower age, gender and interaction frequency with their leader, ethical leadership was positively related to other-praising moral emotions (γ = 0.61, SE = 0.12, *p* < 0.001; Model 1b), supporting hypothesis 1. Meanwhile, the interaction between ethical leadership and leader core self-evaluation was positively related to other-praising moral emotions (γ = 0.18, SE = 0.07, *p* < 0.05; Model 1c), supporting hypothesis 4. Results from Model 2b showed that other-praising moral emotions had a positive effect on reporting unethical issues (γ = 0.41, SE = 0.10, *p* < 0.001), supporting hypothesis 2a. Similarly, results from Model 3b showed that other-praising moral emotions had a negative effect on unethical behavior (γ = −0.02, *SE* = 0.01, *p* < 0.05), supporting hypothesis 2b.

**Table 3:** Hierarchical linear modeling results.

Independent variable	Other-praising moral emotions			Reporting unethical issues		Unethical behavior	
	M1a	M1b	M1b	M2a	M2b	M3a	M3b
Intercept	5.14^∗∗∗^ (0.28)	1.50 (0.78)	2.34^∗∗^ (0.74)	1.51^∗^ (0.57)	0.90 (0.53)	1.50^∗∗∗^ (0.10)	1.52^∗∗∗^ (0.11)
Follower gender	0.14 (0.13)	0.11 (0.13)	0.09 (0.13)	−0.00 (0.15)	−0.01 (0.13)	−0.00 (0.01)	−0.00 (0.01)
Follower age	−0.01 (0.01)	−0.01 (0.01)	−0.01 (0.01)	0.00 (0.01)	0.00 (0.01)	0.00 (0.00)	0.00 (0.00)
Interaction frequency	−0.09 (0.11)	−0.08 (0.11)	−0.10 (0.11)	0.06 (0.09)	0.06 (0.09)	−0.01 (0.01)	−0.01 (0.01)
Ethical leadership		0.61^∗∗∗^ (0.12)	0.56^∗∗∗^ (0.12)	0.58^∗∗∗^ (0.08)	0.33^∗∗^(0.10)	−0.07^∗∗∗^ (0.01)	−0.06^∗∗∗^ (0.01)
Leader core self-evaluation			−0.10 (0.06)				
Ethical leadership ^∗^ Leader core self-evaluation			0.18^∗^ (0.07)				
Other-praising moral emotions					0.41^∗∗∗^ (0.10)		−0.02^∗^ (0.01)
Level 2 *R*^2^	0.00	0.26	0.31	0.52	0.63	0.41	0.40
Level 1 *R*^2^	0.01	0.01	0.01	0.00	0.08	0.01	0.04

*Level 1 n = 289; Level 2 n = 64. Reported coefficients are unstandardized (with robust standard errors). R^2^ is the ratio of intercept variance explained by the level 2 model to the total intercept variance (Hofmann, 1997). ^∗^p < 0.05, ^∗∗^p < 0.01, ^∗∗∗^p < 0.001.*

**FIGURE 2:**
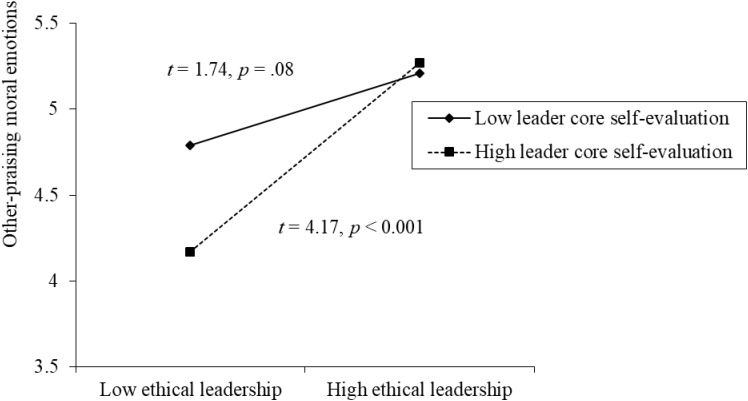
Interaction effect between ethical leadership and leader core self-evaluation on other-praising moral emotions.

We further plotted the interactive effects and performed the simple slop tests. As shown in **Figure 2**, when leader has high core self-evaluation, ethical leadership was significantly positively related to moral emotions (*t* = 4.17, *p* < 0.001). However, when leader core self-evaluation is low, the relationship between ethical leadership and moral emotions was not significant (*t* = 1.74, *p* = 0.08), supporting our hypothesis 4.

We followed Preacher et al. (2010)’s recommendation to use Montel Carlo method to test cross-level indirect effect (i.e., Hypothesis 2a, 2b, 5a, and 5b). Results with 20000 times bootstrapping showed that the indirect effect between ethical leadership and reporting unethical issues via other-praising moral emotions was 0.29, with 95% confidence interval between 0.17 and 0.42 (not including 0), supporting hypothesis 2a. Similarly, the indirect effect between ethical leadership and reporting unethical issues via other-praising moral emotions was −0.02, with 95% confidence interval between −0.024 and −0.011 (not including 0), supporting hypothesis 2b. Moreover, the mediated moderation effect of moral emotions in relationship between hypothesized interaction (i.e., the interaction between ethical leadership and leader core self-evaluation) and reporting unethical issues was 0.08, with 95% confidence interval between 0.06 and 0.10 (not including 0), supporting hypothesis 5a. The mediated moderation effect of moral emotions in relationship between hypothesized interaction and unethical behavior was −0.005, with 95% confidence interval between −0.006 and −0.004 (not including 0), supporting hypothesis 5b.

## Discussion

The present study has investigated how ethical leadership improves followers’ moral actions through generating followers’ moral emotions. We have found that ethical leadership invokes followers’ other-praising moral emotions and eventually promotes moral actions among the followers, such as reporting more ethical conduct and engaging in less unethical behaviors. Furthermore, when ethical leaders have high core self-evaluation, followers’ other-praising moral emotions as well as their subsequent moral actions will be more likely evoked. On the other hand, when the leader has lower core self-evaluation, the positive effect of ethical leadership on follower other-praising moral emotions becomes neutralized.

### Theoretical Contributions

Our research contributes to literature in multiple ways. By introducing the affective perspective, it has offered an emotional explanation about why ethical leadership matters. Previous scholars had consistently suggested that while considering the vital effects of moral emotions on moral actions (Harvey et al., 2016), it is essential for future leadership research to understand the role of moral emotions (Brown and Mitchell, 2010; Lindebaum et al., 2017). For example, followers’ hostile affective states can help explain how followers translate their leader’s mistreatment into their own deviant behaviors (Mayer et al., 2012b). However, although researchers have demonstrated that ethical leadership can benefit followers and teams in multiple ways (Brown et al., 2005; Avey et al., 2012), the question of what role emotions play in employees’ reactions to ethical leadership has not been answered clearly. This answer is important because it helps us understand the causal relationship between ethical leadership and follower moral actions (Brown and Mitchell, 2010) and distinguish ethical leadership from other positive leadership approaches in influencing followers. Drawing on social information processing theory, we have found that ethical leadership evokes followers’ other-praising moral emotions and enhances their moral actions. Specifically, by displaying high moral standards and behaving ethically, ethical leaders invoke follower’s other-praising moral emotions such as elevation, awe, and inspiration, which eventually motivates followers to report more unethical issues and engage in less unethical behavior. Thus, our research has provided an emotional linkage between ethical leadership and follower moral actions, contributing to literature on ethical leadership.

Our research also contributes to emotion literature by focusing on other-praising moral emotions and offering new insights on the association between emotions and behaviors in the moral domain. It is well-documented that emotions have significant effects on individual’s attitudes and behaviors (Cropanzano et al., 2017; Lebel, 2017). However, previous studies have paid more attention on more generalized emotions such as positive emotions and negative emotions (Matta et al., 2014). Although those efforts increased our understanding about how emotions shape individual behaviors, several theorists have argued for more specific-domain research on the differentiated influences of specific emotions on behaviors (Tangney et al., 2007; Brown and Mitchell, 2010; Horberg et al., 2011). For example, several scholars have called for future research to pay attention to moral emotions by revealing its unique role in linking organizational moral standards and employee moral actions (Tangney et al., 2007; Lindebaum et al., 2017). In response to such calls, our research examined the positive association between ethical leadership, follower moral emotions, and moral behaviors. Specifically, our results showed that followers are more likely to generate other-praising moral emotions toward their ethical leaders and then conduct more moral actions, such as reporting more unethical issues. Thus, our research contributes to emotion literature by extending our knowledge about the influence of emotions on behaviors in the moral domain.

Finally, our research has contributed to ethical leadership literature by exploring the boundary conditions under which ethical leaders could be more influential in invoking follower moral emotions. Although the positive impacts of ethical leadership have been examined in previous studies (e.g., Brown et al., 2005; Avey et al., 2012), our knowledge about the conditions under which ethical leadership will be more effective is still far from being satisfactory. Several scholars have noted that without revealing the boundary conditions of effectiveness of ethical leadership, we would not be able reach a comprehensive understanding of ethical leadership (Brown and Treviño, 2006). In response, our results showed that ethical leadership will be more effective when the leader has high core self-evaluation. By contrast, when an ethical leader has low core self-evaluation, followers will not generate other-praising moral emotions toward their leader. Our research thus contributes to ethical leadership literature by investigating how leader characteristics influence the impact of ethical leadership on followers.

### Practical Implications

Our results verify the effectiveness of ethical leadership on follower moral actions, suggesting that ethical leadership is effective to promote employees to behave more ethically. Moreover, our findings show that ethical leaders trigger followers to foster other-praising moral emotions. Organizations should actively hire or cultivate more ethical leaders, since those leaders could benefit followers and, at the same time, the organization. Meanwhile, following our finding that ethical leadership elicits followers’ moral actions, leaders should themselves be more willing to behave ethically. Furthermore, our findings show that when an ethical leader has high core self-evaluation, followers’ moral emotions will be more likely invoked. This result suggests that ethical leaders should be self-motivated and express strong self-confidence in front of their followers. Our result also indicates that when the leader has low core self-evaluation, the positive effects of ethical leadership on followers’ moral emotions will be neutralized. This finding could act as a reminder that ethical leadership may not always be useful.

### Limitations and Future Research

Our study comes with several limitations that should be noted. First, since this study was conducted in China, it is not very clear to what extent can our results be generalized to other contexts. Previous research has indicated that several cultural factors, such as power distance, impact the interactions between leaders and followers (Kirkman et al., 2009). For example, Kirkman et al. (2009) found that the effect of transformational leadership on procedural justice is more positive when followers have low power distance orientation. Similarly, power distance orientation may also affect the relationship between ethical leadership and followers’ moral emotions since followers with different level of power distance orientation may translate their leader’s ethical behavior differently. Thus, we recommend future research examining whether cultural factors make a difference in our proposed model.

Moreover, although our research has revealed the vital role of other-praising moral emotions in linking ethical leadership and follower moral behaviors, we did not exclude the possibility that other kinds of moral emotions may make a difference. For example, as Brown and Mitchell (2010) noted, other-condemning emotions such as disgust and self-focused emotions such as shame may also explain the effect of ethical/unethical leadership on follower behaviors. Meanwhile, in our research, we intended to provide a comprehensive emotional explanation for the influence of ethical leadership on follower moral actions, thus we did not examine whether specific other-praising emotions (e.g., elevation, inspiration, and gratitude) will have distinct effects. Since the behavioral tendency varies across different emotions (Lazarus, 1991), we encourage future research to dig into the emotional link between ethical leadership and follower moral actions.

## Conclusion

The effectiveness of ethical leadership has been well-documented in a growing number of studies. Therefore, it is surprising that we still lack enough knowledge about the emotional linkage between ethical leadership and follower moral actions. The present research proposed and found that ethical leadership prompts followers to engage in more moral actions by invoking followers’ other-praising moral emotions. Moreover, when the ethical leader has high core self-evaluation, the positive effects of ethical leadership on follower moral emotions and moral actions is strengthened. We hope our work will enhance our current knowledge on ethical leadership and provide new insights.

## Ethics Statement

An ethics approval was not required as per our institution’s guidelines and national regulations. Written informed consent was obtained from all participants in our study.

## Author Contributions

YZ and JM designed and adopted the study, wrote the paper. FZ wrote the paper.

## Conflict of Interest Statement

The authors declare that the research was conducted in the absence of any commercial or financial relationships that could be construed as a potential conflict of interest.
